# Applicability of a serodiagnostic line blot for idiopathic inflammatory myopathy: the muscle biopsy is not all

**DOI:** 10.3389/fneur.2024.1504260

**Published:** 2025-01-06

**Authors:** Pedro Nogueira Fontana, Vinícius Gomes da Silva, Roseli Corazzini, Natália Merten Athayde, Ana Marina Dutra Ferreira da Silva, Igor Brockhausen, Carolina da Cunha Correia, Cláudia Ferreira da Rosa Sobreira, Pedro José Tomaselli, Flávio Petean, Rodrigo de Oliveira, Pablo Vinícius Feitoza, Michel Moraes Soane, Natália Saraiva, Rafaela Hidalgo, Cláudia Fideles, David Feder, Alzira Alves de Siqueira Carvalho

**Affiliations:** ^1^Neurosciences and Clinical Department, Centro Universitário ABC, Santo André, Brazil; ^2^Faculty of Medical Sciences, Universidade de Pernambuco, Recife, Brazil; ^3^Department of Neurosciences and Behavioral Sciences, Universidade de São Paulo - Ribeirão Preto, São Paulo, Brazil; ^4^Department of Clinical Surgery, Faculty of Medicine, Universidade Federal do Amazonas, Manaus, Brazil; ^5^EUROIMMUN Brazil, São Caetano do Sul, Brazil

**Keywords:** idiopathic inflammatory myopathies, myositis-specific antibodies, muscle biopsy, dermatomyositis, immune-mediated necrotizing myopathy, anti-synthetase syndrome, inclusion body myositis, myositis-associated antibodies

## Abstract

**Introduction:**

Differential diagnosis of rare idiopathic inflammatory myopathies (IIM) is mainly based on clinical aspects, muscle biopsy analysis, and auxiliary assays that determine myositis-specific and associated autoantibodies (MSA and MAA). While MSAs are considered specific for their respective IIM subclass, MAAs can be present in more than one subclass and in other conditions. This study compares results of a multispecific line blot assay with the final diagnosis of IIM patients based on clinical features and muscle biopsy to draw conclusions for the test's applicability in the diagnostic workflow.

**Methods:**

Samples from patients (*n* = 50) diagnosed with various forms of IIM, including patients (*n* = 5) with other myopathies, were analyzed using the EUROLINE Autoimmune Inflammatory Myopathies 16 Ag (IgG), an anti-HMGCR (IgG) line blot, and the Anti-cN-1A ELISA (IgG, all EUROIMMUN).

**Results:**

MSA and MAA were detected in 74.0% (37/50) of sera and were concordant with the final diagnosis in 64.8% (24/37), discordant in 16.2% (6/37) and not evaluable in 18.9% (7/37) of cases. In 100% (5/5) of patients with other myopathies, no MSA was found. MSA/MAA-co-positivity was observed in 18.0% (9/50) of patients. In 30.0% (15/50) of cases, the muscle biopsy analysis was essential to establish the final diagnosis.

**Conclusion:**

The agreement between serodiagnostic results and final diagnosis highlights the applicability of the EUROIMMUN myositis-related diagnostic test as first line diagnostic tool in the IIM diagnosis workflow and suggests morphological analysis in cases of inconclusive or negative serology. However, results of diagnostic assays shall always be interpreted in combination with clinical features.

## 1 Introduction

Idiopathic inflammatory myopathies (IIM) are rare autoimmune diseases characterized by muscular weakness of varying severity and muscle inflammation. They are highly heterogeneous regarding phenotype, extra muscular involvement, creatine phosphokinase (CK) elevation and association with malignancies (1–3). Usually, the diagnosis is based on clinical aspects, muscle biopsy, and serodiagnostic assays of autoantibodies (AAbs) according to local availability.

The classification of IIM has evolved since the first description in 1975 by Bohan and Peter (4, 5). The advances of immunohistochemistry and the discovery of new myositis-specific antibodies (MSA) at the beginning of the 21st century led to a better understanding of the pathophysiology of IIM (6, 7). MSA have shown relevant clinical associations, e.g., with clinical phenotype, prognosis, risk of malignancy and treatment, providing better management of patients with IIM (8–10). Currently, IIM are divided into immune-mediated necrotizing myopathy (IMNM), dermatomyositis (DM), anti-synthetase syndrome (AsS), inclusion body myositis (IBM) and overlap myositis (OM) (1, 7, 11), based on clinical aspects, morphological findings and myositis-related antibodies [MSA and myositis associated antibodies (MAA)].

**Figure 1:**
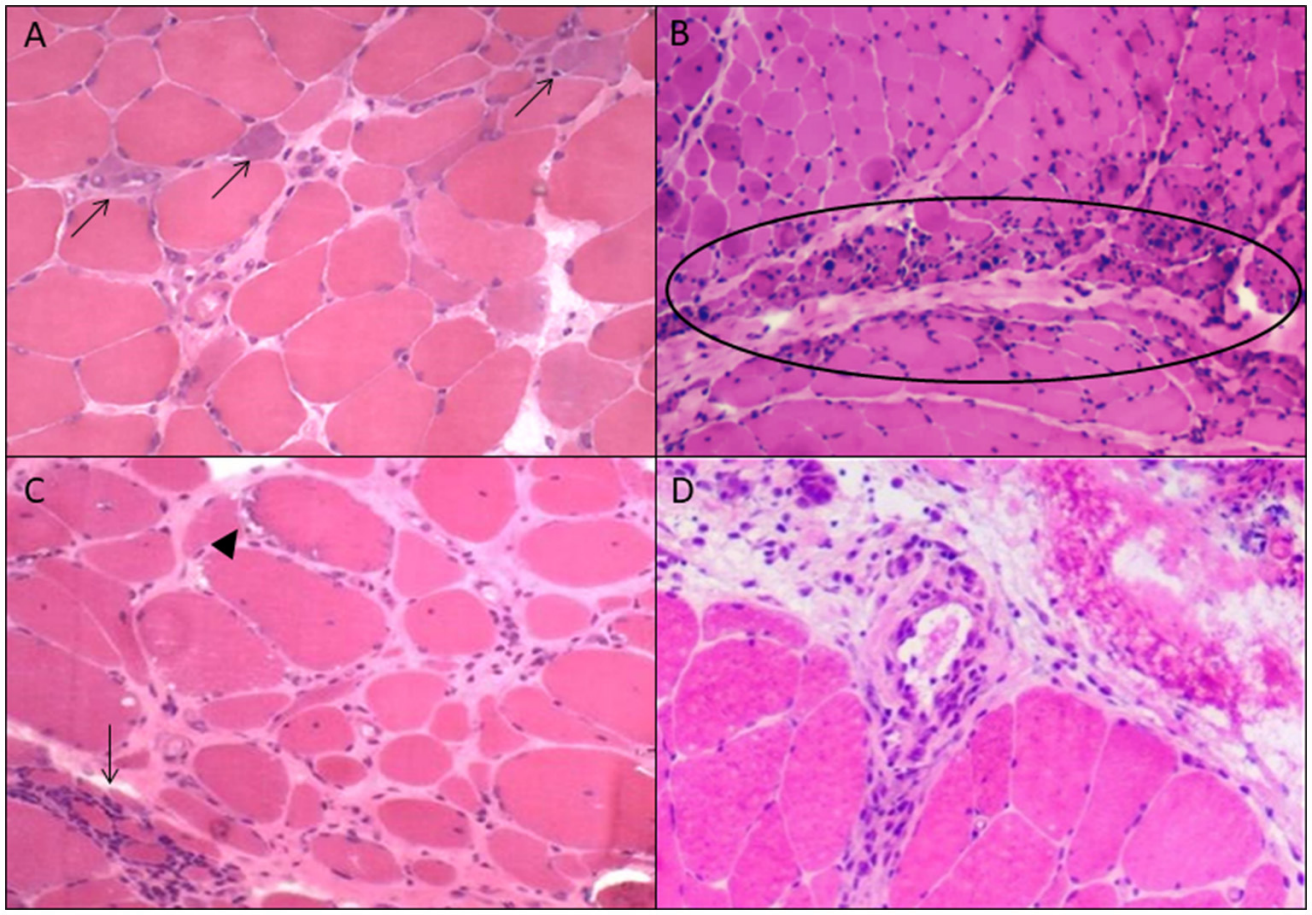
Morphological findings of each idiopathic inflammatory subgroup. **(A)** Immune-mediated necrotizing myopathy: necrosis and regeneration (arrows); **(B)** dermatomyositis: perifascicular atrophy (ellipse); **(C)** inclusion body myositis: rimmed vacuoles (arrowhead) and endomysial inflamation (arrow); **(D)** perimysial myopathy: perimysial inflammation and fragmentation.

Anti-SRP and anti-HMGCR are associated with IMNM. They are related to myocarditis and malignancies, respectively. Also, seronegative cases should be screened for malignancies (12). Anti-Mi-2, anti-TIF1γ, anti-NXP2, anti-MDA5, and anti-SAE are characteristic MSA for DM with anti-TIF1γ, anti-NXP2, and anti-SAE1 related to malignancies (8, 10), and anti-MDA5 associated with severe interstitial lung disease (ILD) (8). Interferon type 1 activation is more distinguished in DM, while in AsS and IBM the interferon type 2 is more important (11). Since 2018, interferon type 1 signature is incorporated in DM diagnostic classification (13). Presence of anti-cN-1A is typical for IBM. However, anti-cN-1A has limited specificity as biomarker (14) because it can be detected in other autoimmune diseases, such as Sjögren's syndrome and systemic lupus erythematosus as well (15), making the muscle biopsy analysis important as diagnostic tool for this subgroup. Finally, AsS is a multisystem disease related to various MSA, such as, anti-Jo-1, anti-OJ, or anti-EJ, frequently in combination with ILD and with different therapeutic targets (16). It can present morphologically as a perimysial myopathy, which can suggest an AsS-specific antibody (Figure 1).

MSA and MAA are detected mainly using immunoprecipitation (IP), ELISA, and line blot assays (17) with ELISA being the most used. IP is gold standard for myositis-related AAb detection applying native antigens. However, it is laborious and requires a high level of expertise, reducing its applicability as a routine test. ELISAs were developed for some antibodies, reaching a high agreement with IP. As new antibodies have been discovered, multispecific assays, such as line blots were developed. They allow easy accessibility and rapid and cost reduced processing but seem less sensitive and less specific (17).

The detection of myositis-related AAbs plays an important role corroborating the final diagnosis in IIM patients. This study aims to evaluate the performance and the clinical applicability of a multispecific line blot accompanied by an anti-HMGCR-specific line blot and an anti-cN-1A-specific ELISA compared to the final diagnosis of suspected IIM patients to draw conclusions for a diagnostic workflow.

## 2 Methods

### 2.1 Patients

This study included patients with IIM based on clinical symptoms and/or muscle biopsy showing typical inflammatory findings. Muscle biopsy samples were obtained according to standard methods (18) and analyzed using histochemistry and immunohistochemistry (IHC) assays. IHC was performed using the antibodies CD4 (Leica Biosystems, clone 4B12), CD8 (Leica Biosystems, clone 4B11), CD20 (Santa Cruz, clone D10 sc-393894), CD68 (Leica Biosystems, clone 514H12), C5b9 (Dako, clone aE11), MHC class I (Dako, clone W6/32) and p62 (Medaysis, clone MD61). The morphology was evaluated according to established criteria (6, 7, 19–22) by two different pathologists blinded to the serological result. Cases in which muscle biopsy analysis showed only MHC-I immunoexpression and no dystrophic changes were classified as immune-mediated myopathy (IMM).

### 2.2 Immunoassays

Patient sera were analyzed using the EUROLINE Autoimmune Inflammatory Myopathies 16 Ag (IgG) comprising the antigens Mi-2α, Mi-2β, -TIF1γ, MDA5, NXP2, SAE1, Ku, PM-Scl100, PM-Scl75, Jo-1, SRP, PL-7, PL-12, EJ, OJ, and Ro-52, a line blot for the detection of anti-HMGCR (IgG), and the anti-cN-1A (IgG) ELISA (all EUROIMMUN Medizinische Labordiagnostika AG, Germany). The assays were performed and evaluated according to the manufacturer's instructions with a sample being positive for presence of an individual antibody at moderate, strong, and very strong line intensities. For the anti-cN-1A (IgG) ELISA, the cut-off at a ratio ≥1.0 was applied. A sample was defined as co-positive when more than one AAb was present. In these cases, the muscle biopsy and clinical findings were considered to corroborate the respective AAb. A sample was defined as discordant when the serologic result differed from the classification based on clinical features and the muscle biopsy (for instance, IBM, DM, IMNM).

### 2.3 Statistical analysis

Fisher's exact test was used to verify the association between gender and serological status. The same test was used for immunosuppressant and serological status. Student's t test was used to verify the association between serological status and age at onset. The Mann-Whitney U test was used to verify the association between serological status and the non-normal quantitative variable CK levels.

### 2.4. Ethics

The study was approved by Clinical Research Ethics Committee of University Center Faculty of Medicine of ABC, CAAE: 49456021.9.0000.0082. Informed consent was obtained from all subjects according to the Declaration of Helsinki.

## 3 Results

### 3.1 Sample characterization

Fifty patients were included, being 18 males and 32 females, aged between 16 and 77 years (mean**:** 50.6 ± 15.7 years). Symptom onset ranged between age of 15–74 years (mean: 47 ± 15.5 years; median: 48 years; Table 1). At time of serum collection, 72% (36/50) of patients were on immunosuppressive therapy. Thirty-one patients were under corticosteroid treatment, 22 were using steroid-sparing agents, seven received intravenous immunoglobulin, one underwent plasmapheresis, one received rituximab and one abatacept.

**Table 1:** Sample characterization.

**Variable**			**Values**
**Demographic data**			
Sex—M:F			18:32
Age (years)			50.6 ± 15.7
Age at symptoms onset (years)			47 ± 15.5
Time interval between symptoms onset and serum collection (years)			2 (1, 5)^*^
Time interval between muscle biopsy and serum collection (years)			0.4 (0; 1.6)^*^
Immunosuppressor use at sample collection moment			36/50 (72%)
**Laboratorial data**			
CK levels (IU/L)			2762 (1,013; 7,147)
**Serological data**			
General positivity			37/50 (74%)
1 antibody			28/50 (56%)
>1 antibody			9/50 (18%)
Anti-Ro52 associated co-positivity			6/9 (66.6%)
IMNM group: positive cases among statin users			6/8 (75%)
	**Positive group (*****n*** = **37)**	**Negative group (*****n*** = **13)**	***p*** **-value**
Male:female ratio	14:23	4:9	0.7
Age at onset (years)	47.4 ± 15.4	46 ± 16.2	0.8
Time between symptoms onset and serum collection (years)	2 (1; 4.3)^*^	2 (1, 8)^*^	0.7
Time between muscle biopsy and serum collection (years)	0.3 (0; 2.0)^*^	1 (0.2; 1.4)^*^	0.2
CK levels (IU/L)	3,077 (989.5; 7,147)^*^	2,200 (1,222; 8,537)^*^	0.99
Immunosuppressant use at sample collection moment	27 (72.9%)	9 (69.2%)	0.4

^*^Median (percentile 25%; percentile 75%).

M, male; F, female; CK, creatine phosphokinase.

### 3.2. Diagnosis according to clinic, histochemistry and immunohistochemistry

According to clinical picture and analysis of the muscle biopsy, 50 patients were diagnosed with IIM. The CK concentration in sera of these patients varied between 50 and 19,459 IU/L at a value considered as normal below 170 IU/L. In detail, IMNM in 21/50 (42%), of which eight were under statin use, DM in 8/50 (16%), immune-mediated myopathy (IMM) in 7/50 (14%), IBM in 5/50 (10%), and perimysial myopathy (PMM) in 4/50 (8%) patients. In five of 50 patients (10%), of which four were under statin use, other non-autoimmune mediated myopathies were found (cytoplasmic body myopathy, lipid storage myopathy, protein aggregate myopathy, nemaline myopathy and calpainopathy, Table 2).

**Table 2:** Frequency of morphological and serological findings; sensitivity/specificity/PPV/NPV.

**Morphological findings**	***n* = 50**	**Positivity**	**MSA**	**Sens**	**Spec**	**PPV**	**NPV**
IMNM	21 (42%)	17 (81%)	SRP/HMGCR	71.4%	82.7%	75%	80%
DM	8 (16%)	6 (75%)	Mi-2/NXP2/SAE1	50%	90.4%	50%	90.4%
IBM	5 (10%)	3 (60%)	cN-1A	60%	91.1%	42.8%	50%
PMM	4 (8%)	4 (100%)	Jo-1, OJ	50%	95.6%	50%	95.6%
IMM	7 (14%)	6 (85.7%)	—^a^	—	—	—	—
Other^b^	5 (10%)	1 (20%)	—^c^	—	—	—	—

^a^1 case positive for SRP, 1 for Ku, one for cN-1A, 1 for Jo-1+Ro52, 1 for MDA5 + PL7 + HMGCR, 1 for cN-1A + SRP and 1 seronegative.

^b^1 cytoplasmic body myopathy, 1 lipid storage myopathy, 1 protein aggregate myopathy, 1 nemaline myopathy and 1 calpainopathy.

^c^4 seronegatives cases and one positive for Ro52.

MSA, myositis-specific antibodies; Sens, sensitivity; Spec, specificity; PPV, positive predictive value; NPV, negative predictive value; IMNM, immune-mediated necrotizing myopathy; DM, dermatomyositis; IBM, inclusion body myositis; PMM, perimysial myopathy; IMM, immune-mediated myopathy.

### 3.3. Serodiagnostics of MSA and MAA

In 37/50 sera (74.0%), MSAs (*n* = 34, 91.9% of positive samples) and MAAs (*n* = 3, 8.1%) were present. In 28 cases, sera were positive for one individual antibody; nine sera showed co-positivity, being five cases MSA-MAA copositivity and four MSA-MSA copositivity (Supplementary Table 1).

In the IMNM panel, sera were positive for anti-HMGCR and anti-SRP in 6/21 and 7/21 samples, respectively. From eight IMNM patients under statin use, five were positive for anti-HMGCR and one for anti-SRP. Moreover, one sample each was positive for anti-Mi-2, anti-Ku, co-positive for anti-SRP/anti-Ro52, and co-positive for anti-cN-1A/anti-HMGCR/anti-Ro52. Four samples were negative for the MSAs and MAAs investigated here. In DM sera, one sample each was positive for anti-SRP, anti-Mi-2, anti-NXP2, anti-cN-1A, co-positive for anti-SAE1/anti-Ro52/anti-Ku, and co-positive for anti-SAE1/anti-Ro52 and two samples were negative for MSA and MAA. From the PMM patients, one each was positive for anti-HMGCR and anti-Mi-2 and two sera were positive for anti-Jo-1. In IMM patient sera, in one sample each anti-SRP, anti-Ku, anti-cN-1A, anti-Jo-1/Ro52, anti-MDA5/anti-PL7/anti-HMGCR, and anti-cN-1A/anti-SRP were detected. One sample was negative for MSA and MAA. Sera from patients with IBM were positive for anti-cN-1A, anti-cN-1A/Ro52, and anti-cN-1A/anti-SRP with two samples negative for the MSA and MAA investigated here.

In sera from patients diagnosed with other non-immune mediated myopathies, one sample was anti-Ro52 positive, all other samples were seronegative (Supplementary Table 1). Agreement between muscle biopsy and serology occurred in 64.8% (24/37). Based on sub-class characteristic MSA and MAA, IMNM, DM, IBM, and PMM were found with a sensitivity of 71.4% (15/21), 50.0% (4/8), 60.0% (3/5), and 50.0% (2/4) and a specificity of 82.7%, 90.4%, 91.1%, and 95.6%, respectively (Table 2, Supplementary Table 3). In 6/37 (16.2%) cases, the results were discordant (Supplementary Table 2). In 7/37 (18.9%) sera from patients with IMM, concordance or discordance could not be determined since a specific AAb could not be defined for this myositis subclass. In 5/5 (100.0%) sera from patients with other myopathies, no MSA was found (Table 2).

## 4 Discussion

This study describes the results of a cohort of patients with IIM using muscle biopsy associated with clinical features as the gold standard for diagnosis in order to compare them with serological results (MSA/MAA).

There is some disagreement between different authors about which antibodies should be considered myositis-specific or myositis-associated (23); however, ~70% of IIM patient carry some myositis-related antibody (24). In the present study, we found a general positivity of 74% (37/50) and a MSA positivity of 83.8% (31/37), similar to what was previously reported (25). The high prevalence of immunossuppressive treatment (72%) suggests that it did not influence the serological results. According our results, even in patients with long standing disease, the serology test can be useful.

**Figure 2:**
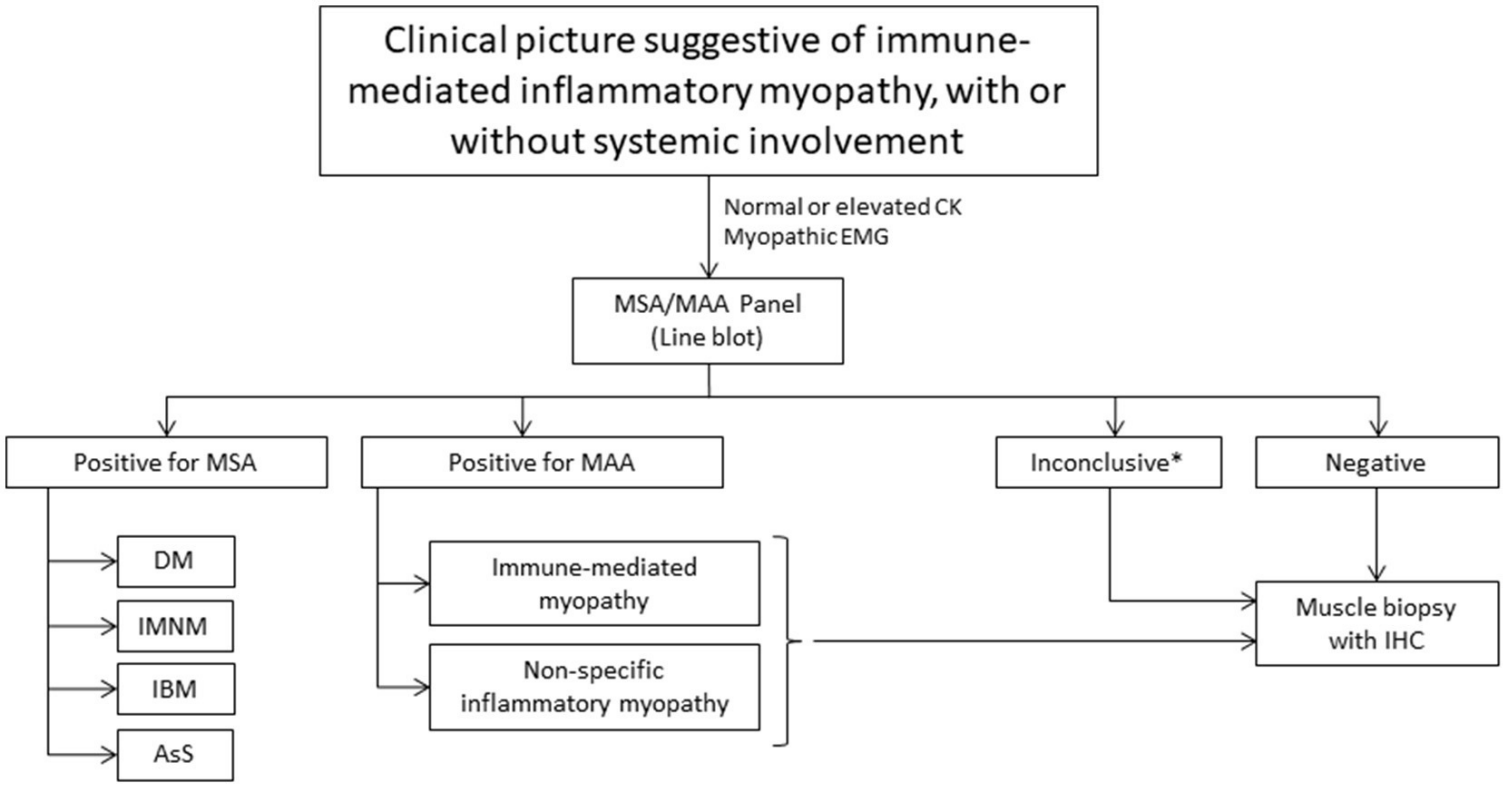
Diagnostic flowchart. CK, creatine phosphokinase; MSA, myositis-specific antibodies; MAA, myositis-associated antibodies; DM, dermatomyositis; IMNM, immune-mediated necrotizing myopathy; IBM, inclusion body myositis, AsS, anti-synthetase syndrome; IHC, immunohistochemistry. *Copositivity cases or clinical-serological discordance.

Although the sensitivity of MSA/MAA is limited, our study showed a good specificity, suggesting that MSA/MAA panel is a useful tool in the diagnostic workup (Table 2 and Figure 2). Furthermore, in cases in which the muscle biopsy showed findings compatible with some subtypes of IIM but with no evident clinical features compatible, the identification of AAbs helped the diagnostic conclusion. In the P13, with anti-Ku positivity and compatible clinical syndrome, the muscle biopsy showed IMNM aspects. In this case, anti-Ku defined the diagnosis. This is in accordance with previous reports showing that IMNM can occur in other diseases as anti-Ku syndrome (26).

Our findings suggest that MSA/MAA panel is a useful diagnostic tool, in accordance with previously described (1, 6, 27, 28). The performance of multispecific line blot panels, being less invasive compared to muscle biopsy, makes this method an important ally as initial testing in the diagnostic evaluation of IIM. Our study, for the first time, showed 64.8% agreement between muscle biopsy findings and presence of antibodies, suggesting that the serological test can be used as a screening in IIM in order to avoid an invasive procedure. This high positivity led us to propose a diagnostic flowchart in IIM (Figure 2). Furthermore, the associations between MSA/MAA and clinical characteristics can guide specific therapeutic approaches, including malignancy screening and early management of ILD.

Malignancies are a factor of worse prognosis in IIM (29, 30). Classically, TIF1γ, NXP2 and SAE1 antibodies delimit a subset of subjects under a high risk of cancer, indicating the need for neoplastic screening and close monitoring, especially in the first 3 years after the onset of symptoms (27, 31–41). Although anti-HMGCR has been considered associated with neoplasia, some recent studies concluded the opposite and further investigations are necessary (42–44). Furthermore, seronegative IMNM subjects also are at higher risk of malignancies, which highlights the importance of serodiagnostics in this group of IIM (32). This predictive value is an advantage over muscle biopsy that, in general, cannot suggest a malignancy association. An exception to this are the punched out fibers in adult DM, found in TIF1γ cases (27); this pathological feature is particularly important in DM, since immunoblot have up to 50% of false-negativity for TIF1γ, and makes muscle biopsy important in seronegative DM (45). In addition, necrosis and regeneration, suggestive of IMNM, can be present in different neuromuscular disorders, so the presence of antibodies can guide the diagnostic approach.

Another important aspect of serology in DM subgroup is morphological similarity that can occur in both DM and AsS although with different pathophysiological mechanisms (46). The Mi2 positivity in one of our cases of perimysial myopathy (P31; Supplementary Table 2) is an example of this similarity. Also, in IMNM, serological diagnosis can help guide therapy, as individuals with refractory SRP positivity IMNM may have a good response to rituximab (19, 47, 48). In this context, MSA/MAA panels help to accelerate the diagnosis of IIM with a specific therapeutic target in a non-invasive way, allowing an early treatment.

The discovery of the role of interferon I and II in IIM (49) and the therapeutic effect of JAK/STAT inhibitor ruxolitinib in DM (9) brought new insights into the pathophysiology of IIM. In DM, there is an overexpression of genes induced by type I interferon (50, 51), making it a potential therapeutic target in this disease and paving the way for target-specific treatment.

Two cases were defined based on clinical features and serology but not by muscle biopsy (P24 and P48). The presence of eyelid rash and arthralgia (P24) along with Jo-1 allowed the diagnosis of AsS; although eyelid rash is considered typical of DM, it also can occur in AsS (52). A case with both SRP and cN1A (P48) could not be defined on pathological basis, but clinical features and CK levels were suggestive of IBM and in agreement with cN1A result (Supplementary Table 2).

In 15 (30%) cases (Supplementary Table 2), the muscle biopsy and clinical features were needed to define the final diagnosis, suggesting that the serology alone may not replace the clinical diagnosis and muscle biopsy. Disagreement between muscle biopsy and serology occurred in 16.2%. These cases had the final diagnosis defined by clinical features and muscle biopsy, suggesting that MSA can be found in different IIM; therefore, serology not always will determine the final diagnosis (Table 2), but is important as auxiliary diagnostic tool. Several reasons could explain this biopsy-antibody discordance, suggesting that serological tests should be used as screening or when the diagnostic workup was not conclusive. The different techniques available to detect AAbs in IIM can lead to different results (17, 19). The main methods used for AAbs detection are line blot, immunoprecipitation (IP) and ELISA (17), the former being the most used. IP, a conventional technology considered as a gold-standard for MSA and MAA detection, is laborious and require a high level of expertise, reducing its availability as a routine test. Thus, ELISA was developed for some antibodies, reaching a high agreement with IP. As new antibodies have been discovered, multispecific assays were developed, in order to be more accessible and cost reduced, although with lesser sensitivity and specificity (17). In the case of line blot, a multispecific assay, previous studies indicated that adjusting cutoff levels may be needed to increase specificity (53–55). Although imunoblot method has some limitations when compared to IP, several studies have demonstrated its usefulness in the current clinical practice as a screening tool (56, 57).

Co-positive cases represented a challenge to serological diagnosis of IIM. In 12% of cases, the responsible antibody was based on muscle biopsy diagnosis. Therefore, the muscle biopsy should not be the first exam in diagnostic workup of IIM. Nevertheless, serological testing remain useful in determine the prognosis and clinical approach in IIM.

This study shows a high agreement rate between serology (MSA/MSA) and morphological data, highlighting the importance and applicability of antibody testing as a screening test in IIM, being the muscle biopsy performed when serology is negative. Only in these cases, as well as in discordant cases, IP should be applied, as the higher costs and methodological issues limit its availability in the current clinical practice. Moreover, the serology is useful in determining prognosis and therapeutic approach. A limiting point of this study is the small sample size and, consequently, the low number of cases in some subgroups, common in rare diseases, which limits the interpretation of the results. A more robust sample would help to confirm our results, since a joint analysis of clinic, specific serology and muscle biopsy is not the daily reality of many services, and sometimes the patient is treated with an incomplete investigation. Nevertheless, our results show that clinical features remain the most important key point to reach the final diagnosis, regardless of isolated laboratorial tests.

## Data Availability

The original contributions presented in the study are included in the article/Supplementary material, further inquiries can be directed to the corresponding author.
