# Corrigendum: Applicability of a serodiagnostic line blot for idiopathic inflammatory myopathy: the muscle biopsy is not all

**DOI:** 10.3389/fneur.2025.1567315

**Published:** 2025-02-12

**Authors:** Pedro Nogueira Fontana, Vinícius Gomes da Silva, Roseli Corazzini, Natália Merten Athayde, Ana Marina Dutra Ferreira da Silva, Igor Brockhausen, Carolina da Cunha Correia, Cláudia Ferreira da Rosa Sobreira, Pedro José Tomaselli, Flávio Petean, Rodrigo de Oliveira, Pablo Vinícius Feitoza, Michel Moraes Soane, Natália Saraiva, Rafaela Hidalgo, Cláudia Fideles, David Feder, Alzira Alves de Siqueira Carvalho

**Affiliations:** ^1^Neurosciences and Clinical Department, Centro Universitário ABC, Santo André, Brazil; ^2^Faculty of Medical Sciences, Universidade de Pernambuco, Recife, Brazil; ^3^Department of Neurosciences and Behavioral Sciences, Universidade de São Paulo - Ribeirão Preto, São Paulo, Brazil; ^4^Department of Clinical Surgery, Faculty of Medicine, Universidade Federal do Amazonas, Manaus, Brazil; ^5^EUROIMMUN Brazil, São Caetano do Sul, Brazil

**Keywords:** idiopathic inflammatory myopathies, myositis-specific antibodies, muscle biopsy, dermatomyositis, immune-mediated necrotizing myopathy, anti-synthetase syndrome, inclusion body myositis, myositis-associated antibodies

In the published article, an author name was incorrectly written as Pedro Tomaselli. The correct spelling is Pedro José Tomaselli.

The authors apologize for this error and state that this does not change the scientific conclusions of the article in any way. The original article has been updated.

