# Supplementary material for: Applicability of a serodiagnostic line blot for idiopathic inflammatory myopathy: the muscle biopsy is not all
Source: Front Neurol. 2025 Jan 6;15:1504260. doi: 10.3389/fneur.2024.1504260 (PMC11743459; doi:10.3389/fneur.2024.1504260)
Supplement: Supplementary file 1 [file Table_1.docx]

Supplementary table 1: Morphological and serological data

| Antibodies | | Morphological diagnosis (muscle biopsy) | | | | | | Total |
| --- | --- | --- | --- | --- | --- | --- | --- | --- |
|  |  | IMNM | DM | PMM | IMM | IBM | Others |  |
| MSA | SRP | 7 | 1* | - | 1 | - | - | 9 |
|  | HMGCR | 6 | - | 1* | - | - | - | 7 |
|  | Mi2 | 1* | 1 | 1* | - | - | - | 3 |
|  | NXP2 | - | 1 | - | - | - | - | 1 |
|  | cN1A |  | 1* | - | 1 | 1 | - | 3 |
|  | Jo1 | - | - | 2 | - | - | - | 2 |
| MAA | Ku | 1* | - | - | 1 | - | - | 2 |
|  | Ro52 | - | - | - | - | - | 1 | 1 |
| MSA+MAA | SRP/Ro52 | 1 | - | - | - | - | - | 1 |
|  | SAE1/Ro52/Ku | - | 1 | - | - | - | - | 1 |
|  | Jo1/Ro52 | - | - | - | 1 | - | - | 1 |
|  | cN1A/Ro52 | - | - | - | - | 1 | - | 1 |
|  | SAE1/Ro52 | - | 1 | - | - | - | - | 1 |
| MSA+MSA | MDA5/PL7/HMGCR | - | - | - | 1 | - | - | 1 |
|  | cN1A/HMGCR/Ro52 | 1 | - | - | - | - | - | 1 |
|  | cN1A/Mi2 | - | - | - | - | 1 | - | 1 |
|  | cN1A/SRP | - | - | - | 1 | - | - | 1 |
|  | Seronegatives | 4 | 2 | - | 1 | 2 | 4 | 13 |
|  | Total | 21 (42%) | 8 (16%) | 4 (8%) | 7 (14%) | 5 (10%) | 5 (10%) | 50 |

Legend – MSA: myositis-specific antibody; MAA: myositis-associated antibody; IMNM: immune-mediated necrotizing myopathy; DM: dermatomyositis; PMM: perimysial myopathy; IMM: immune-mediated myositis; IBM: inclusion body myositis; *Discordance between muscle biopsy and serology
