# Supplementary material for: Applicability of a serodiagnostic line blot for idiopathic inflammatory myopathy: the muscle biopsy is not all
Source: Front Neurol. 2025 Jan 6;15:1504260. doi: 10.3389/fneur.2024.1504260 (PMC11743459; doi:10.3389/fneur.2024.1504260)
Supplement: Supplementary file 2 [file Table_2.docx]

Supplementary table 2: Demographic, clinical and laboratorial data of copositive and discordant cases

| Case | Gender | Age | CK | Positivity  (band intensity or ELISA titer^a^) | Morphological diagnosis | Clinical features |
| --- | --- | --- | --- | --- | --- | --- |
|  |  |  |  | Copositive cases |  |  |
| P01 | M | 47 | 211 | **SAE1** (73)  Ro52 (82)  Ku (11)^b^ | DM | Cerv and prox Wk (UL>LL), dysp, shawl sign, facial eryth |
| P11 | F | 67 | 50 | **SAE1** (47)  Ro52 (11)^b^ | DM | Dysp, ILD, facial ulcers and plaques |
| P14 | F | 27 | 5900 | **HMGCR** (18)  Ro52 (35)  cN1A (1,07)^b^ | IMNM | Arthr, mlg, and prox Wk, SLE |
| P15 | M | 50 | 5026 | **SRP** (104)  Ro52 (79) | IMNM | Weight loss, prox Wk, gait with bilateral support, dysp |
| P22 | M | 47 | 3077 | **cN1A** (4,09)  Ro52 (45) | IBM | Arthr, prox and distal Wk, fist sign, dry eye |
| P24 | F | 32 | 19000 | **Jo1 (64)**  Ro52 (87) | IMM | Arthr, mlg, violaceous eyelid lesions^c^ |
| P34 | F | 77 | 196 | **cN1A** (5,34)  Mi2 (12)^b^ | IBM | Falls, prox Wk in LL and distal Wk in UL, loss of gait, dysp and dry mouth |
| P46 | F | 57 | 1360 | MDA5 (15)^b^  PL7 (13)^b^  HMGCR (11)^b^ | IMM | Mlg, arthr, fatigue and prox Wk, mucous membrane pemphigoid^d^ |
| P48 | F | 64 | 680 | **cN1A** (2,41)  SRP (18) | IMM | Slowly progressive prox and distal Wk and dysph^e^ |
|  |  |  |  | Discordant antibodies |  |  |
| P07 | M | 47 | 147 | SRP (14)^b^ | DM | Prox Wk, dysp, facial eryth, hands and back |
| P13 | F | 51 | 1159 | Ku (89) | IMNM | Arthr and odynophagia, Also positive for anti-Scl70, anti-RNP, anti-peroxidase |
| P16 | F | 26 | 1767 | HMGCR (20) | PMM | Prox Wk, dysp, Rayn, arthritis, oral and genital ulcer, knotty skin lesions |
| P17 | F | 40 | 1151 | cN1A (3,37) | DM | Prox Wk, weight loss, dysph, pleural effusion, Rayn, scaly facial eryth |
| P31 | M | 19 | 620 | Mi2 (13)^b^ | PMM | Prox Wk, antecubital and thorax eryth |
| P45 | M | 38 | 17000 | Mi2 (14)^b^ | IMNM | Generalized Wk (prox predominance) and dysp; Hypothyroidism |

Legend: Bold antibodies are compatible with the clinical picture and muscle biopsy; ^a^cN1A: ELISA; all other antibodies: line blot assay. ^b^antibodies near the lower range of positivity; ^c^probable anti-synthetase syndrome based on skin changes and serology; ^d^Not possible to define the diagnosis; ^e^Probable IBM based on clinical features and serology; cerv: cervical; prox: proximal; Wk: weakness; UL>LL: upper limbs weaker than upper limbs; dysp: dysphagia; ILD: interstitial lung disease; arthr: arthralgia; LL: lower limbs; UL: upper limbs; Rayn: Raynaud’s phenomenon; eryth: erythema; mlg: myalgia; SLE: systemic lupus erythematosus.
