# Supplementary material for: Applicability of a serodiagnostic line blot for idiopathic inflammatory myopathy: the muscle biopsy is not all
Source: Front Neurol. 2025 Jan 6;15:1504260. doi: 10.3389/fneur.2024.1504260 (PMC11743459; doi:10.3389/fneur.2024.1504260)
Supplement: Supplementary file 3 [file Table_3.docx]

Supplementary Table 3: distribution of positive and negative cases according to subgroup

1. Immune-mediated necrotizing myopathy: SRP and HMGCR

|  | IMNM cases | Non IMNM cases | Total |
| --- | --- | --- | --- |
| Positives | 15 | 5 | 20 |
| Negatives | 6 | 24 | 30 |
| Total | 21 | 29 | 50 |

Sensitivity: 71,4%

Specificity: 82,7%

Positive predictive value: 75%

Negative predictive value: 80%

2) Dermatomyositis: Mi2, NXP2, TIF1γ, MDA5, SAE

|  | DM Cases | Non DM cases | Total |
| --- | --- | --- | --- |
| Positives | 4 | 4 | 8 |
| Negatives | 4 | 38 | 42 |
| Total | 8 | 42 | 50 |

Sensitivity: 50%

Specificity: 90,4%

Positive predictive value: 50%

Negative predictive value: 90,4%

3) Inclusion Body Myositis

|  | IBM Cases | Non IBM Cases | Total |
| --- | --- | --- | --- |
| Positives | 3 | 4 | 7 |
| Negatives | 2 | 41 | 43 |
| Total | 5 | 45 | 50 |

Sensitivity: 60%

Specificity: 91,1%

Positive predictive value: 42,8%

Negative predictive value: 50%

4) Perimysial myopathy: Jo1, PL7, PL12, EJ, OJ

|  | PMM Cases | Non PMM Cases | Total |
| --- | --- | --- | --- |
| Positives | 2 | 2 | 4 |
| Negatives | 2 | 44 | 46 |
| Total | 4 | 46 | 50 |

Sensitivity: 50%

Specificity: 95,6%

Positive predictive value: 50%

Negative predictive value: 95,6%
